# The Clinical Value of Poor-Law Infirmaries: The Root of the Medical Difficulty

**Published:** 1915-02-20

**Authors:** 


### THE CLINICAL VALUE OF POOR-LAW INFIRMARIES.

### The Root of the Medical Difficulty.

By A FORMER MEDICAL SUPERINTENDENT.

Virtue proverbially attaches to the "last word." In the recent discussion in the columns of The Hospital concerning "The Clinical Value of Poor-Law Infirmaries," the writer of the first article (published in the number of January 23) has hit squarely on the head a nail which had already been driven nearly home when he says: "As an infirmary officer I was overworked, nearly run off my feet, and could never find time to do the work as thoroughly and as conscientiously as I would have liked." At the root of infirmary medical difficulties lies shortage of medical staff.

No class of institution has developed as has the Poor-Law infirmary; no class of institution has been so starved as regards its staff: it is to the Metropolitan and neighbouring infirmaries that these remarks especially apply. The infirmary medical superintendent of to-day often finds himself with the sick for whom infirmaries were originally intended in wards in the workhouse, and, also on his hands in the infirmary, a mass of patients who would, forty years ago, have never entered a Poor-Law institution. The beds in a progressive infirmary are no longer occupied by the cast-off patients of the hospitals, by the destitute suffering from chronic bronchitis, osteo-arthritis, ulceration of the leg, and spinal-cord sclerosis. The Poor-Law Guardian remains much what he was, as often a Guardian of the rates as a Guardian of the poor; his idea of medical treatment is the taking of medicine, and his knowledge of the surgical and gynæcological revolution which has occurred during his lifetime is hazy in the extreme. He seldom attempts to compare the work of an infirmary staff with that of the numerous and specialised staff of a general hospital. He may sometimes talk glibly of "conducting the infirmary on hospital lines," but of the needs of those lines he knows nothing; he may yearn for development, but is averse to spending money upon it.

The general hospital and the infirmary are essentially different, especially when to the former a medical school is attached. It is needless here to refer at length to the duties and responsibilities of the infirmary medical superintendent; it suffices to say that all he can hope to do is to make his infirmary successful. He can gain success in that he is in a position to afford to nine out of ten of his patients treatment as effectual as they could obtain in a hospital. He can control an excellent infirmary, but, should he attempt all-round competition with the general hospital, he will court, and will at many points meet, disappointment and disillusionment.

To a limited extent only can the infirmary medical superintendent "justify the existence of his institution by records of clinical and scientific value." Clinical records must primarily be those which are of value in treatment; their scientific value is, or should be, even in a teaching institution, a secondary consideration. The importance and requirements of medico-legal records and evidence are alike, and similarly concern medical men, whether in institutions or in private practice. The making of clinical notes and records in infirmaries is largely the duty of assistant medical officers. Only those to whom infirmary work is well known have any conception of the difficulty which has for years past been found in getting (even with a slow but steady increase of salaries) keen, loyal, and competent men, and of the constant changes and temporary stop-gaps in the assistant staff. The conditions militate against efficiency and against the keeping of continuous records. The time has not yet come when the value of the work of the infirmary can be adequately judged by the value of the records of its scientific work.

If there be contempt for infirmary medical work, it is in the opinion of those who, ignorant of the conditions, expect a machinery and organisation which does not yet exist. The idea that "the absence of clinical records . . . to some extent justifies the prejudice of a few poor against the rate-aided institution" is absurd. The "few" know and care nothing about clinical records, *post-mortem* or otherwise; such prejudice as remains is against the association with "paupers" and "the workhouse," against the "rate-aid," and not the institution.

[Further correspondence upon this subject will be found on page 469.—Ed. The Hospital.]

